# A Building‐Integrated Hybrid Photovoltaic‐Thermal (PV‐T) Window for Synergistic Light Management, Electricity and Heat Generation

**DOI:** 10.1002/advs.202408057

**Published:** 2024-11-25

**Authors:** Francesco Polito, Gan Huang, Christos N. Markides

**Affiliations:** ^1^ Clean Energy Processes (CEP) Laboratory Department of Chemical Engineering Imperial College London SW7 2AZ London UK; ^2^ Institute of Microstructure Technology Karlsruhe Institute of Technology Hermann‐von‐Helmholtz‐Platz 1 76344 Eggenstein‐Leopoldshafen Germany

**Keywords:** building‐integrated, photovoltaics, photovoltaic‐thermal, PV‐T, renewables, solar

## Abstract

The installation of common solar panels and collectors in the built environment requires access to significant roof space, which is limited. This motivates the development of high‐efficiency, building‐integrated technologies that can maximize space utilization and energy provision. In this work, a building‐integrated hybrid photovoltaic‐thermal window (PVTW) is fabricated and tested, composed of a semi‐transparent photovoltaic (PV) layer and a selectively absorptive liquid‐based thermal absorber. It is demonstrated that, at 30° inclination, the PVTW can simultaneously generate electricity, with an electrical efficiency of 3.6%, and provide ≈50 °C water, with a thermal efficiency of 10.7%, in the middle of a typical summer day (20th July) in London (maximum ambient temperature ≈34 °C, solar irradiance ≈1100 W m^−2^ at midday). The water temperature decreases by ≈7 °C, whilst thermal efficiency improves to 17.6% as the inclination angle increases to 90° (vertical); the electrical efficiency reduces marginally (3.3%). Compared to a liquid‐based solar‐thermal window (STW), the PVTW can generate hot water at ≈10 °C higher temperature and with 10% absolute increase in thermal efficiency when the inclination angle is 60°, plus electricity. The wider uptake of this technology in glass‐based urban spaces has the potential to generate significant energy while reducing building temperature management costs.

## Graphical abstract

A building‐integrated hybrid photovoltaic‐thermal (PV‐T) window prototype is designed, fabricated and tested for simultaneous light management, heat and electricity production. Such a solution has the potential to exploit the ever‐growing use of glass as a building material in the urban environment, therefore tackling horizontal space limitations and abating the distance between generation and consumption of energy.

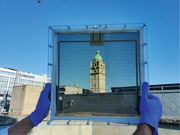

## 1 Introduction

Around 170 PW of solar energy continuously reaches the earth's surface,^[^ ^1^ ^]^ which can be harvested and used to generate electricity, via photovoltaic (PV) panels, or to provide heat or hot water, via solar‐thermal (ST) collectors.^[^ ^2^ ^]^ One of the unique advantages of these–nowadays common–solar technologies is their excellent suitability to distributed applications, and the possibility, via urban integration, to reduce the distance between generation and consumption, therefore abating transmission/transportation, and other infrastructure losses and expenses.^[^ ^3^ ^]^ It also alleviates the demands for land allocation typically associated with utility‐scale solar farms.^[^ ^4^ ^]^

The past 30 years have hence seen a growing interest in the allocation of both PV and ST systems within the urban environment.^[^ ^5^ ^]^ Initial deployment of solar collectors in buildings occurred on rooftops, exploiting horizontal or sloped surfaces. The literature presents a number of relevant examples and studies, however, these mainly consider the impact of atmospheric conditions on performance, for example, performance under different solar conditions, or how increased wind speed and reduced ambient temperatures can affect PV devices.^[^ ^6^ ^]^ Subsequently, vertical surfaces started being exploited via covering façades with PV panels or ST collectors as reported in several reviews.^[^ ^7^ ^]^ The different characteristics of PV and ST technologies lead to opposite design requirements. PV panels benefit from cooling, which can be promoted, e.g., by the addition of air channels between the panel and the façade, whereas ST absorbers are designed for thermal accumulation, making insulation desirable.^[^ ^8^ ^]^ Therefore, a promising design avenue for building‐integrated PV systems involves redirecting the hot air generated from PV panel cooling to building interiors, which helps to reduce the building's heating demand. Numerous investigations have been conducted with the aim of optimizing such (and, other) designs, covering aspects from integration mechanism,^[^ ^9^ ^]^ material selection,^[^ ^10^ ^]^ system operation,^[^ ^11^ ^]^ performance under dynamic conditions,^[^ ^12^ ^]^ and domestic applications.^[^ ^13^ ^]^

The more recent development of semi‐transparent PV cells has paved the way to new building integration possibilities.^[^ ^14^ ^]^ Cadmium telluride (CdTe), organic films, perovskites, and amorphous silicon (a‐Si) are among the most promising semi‐transparent PV materials. Thanks to window integration, glass‐based sections of buildings can now be exploited for power generation.^[^ ^15^ ^]^ Skyscrapers, with their vast vertical glass surfaces, are an appropriate target for this technology, which can generate electricity and help reduce the heating demand,^[^ ^16^ ^]^ while also improving the habitability of interior spaces via light management, thus filtering harmful ultraviolet (UV) radiation.^[^ ^17^ ^]^

The history of semi‐transparent PV windows is recent, with only few examples of this technology and almost no commercially available products. Different approaches have been used to achieve transparency. One method involves the use of materials with specific optical properties, i.e., transparent to a portion of the visible light, while also being photo‐active (for electricity generation) to another portion of the solar spectrum. Polysolar's “Transparent Solar Glass” is an example of such a technology based on a‐Si,^[^ ^18^ ^]^ and testing on this PV‐glass has been conducted.^[^ ^19^ ^]^ Yet, despite its encouraging technical performance, commercialization is still lagging for this technology.^[^ ^20^ ^]^ Another proposed solution is the employment of luminescent solar concentrators; these are materials capable of diverting a portion of the spectrum toward the device's frame where traditional PV material is deposited. Such an approach is being explored by companies such as UbiQD,^[^ ^21^ ^]^ Physee Solar,^[^ ^22^ ^]^ and Glass to Power,^[^ ^23^ ^]^ as well as in a study by Meinardi et al.^[^ ^24^ ^]^ A further approach involves the use of opaque PV materials, which only partially cover the panel surface (e.g., a set of spaced PV stripes or cells). Such panels are being developed by companies like Onyx Solar,^[^ ^25^ ^]^ Brite,^[^ ^26^ ^]^ and Polysolar.^[^ ^27^ ^]^ Most early designs involved macroscopic PV cells (order of cm) separated by like‐sized stripes (gaps) of transparent material.^[^ ^28^ ^]^ More recently, designs have attempted to tune visibility by miniaturizing the cells and gaps so to be only visible over short distances, giving the product an overall translucent dark appearance.^[^ ^29^ ^]^

Despite the less challenging material requirements, semi‐transparent thermal absorbers have also only recently attracted the interest of academia and industry. Initially, airflows within window cavities were employed to manipulate the internal temperature of buildings, but subsequently, water was employed for its improved properties.^[^ ^2^ ^]^ Recent efforts are focusing on further improving the thermophysical properties of the fluid via nanoparticle doping,^[^ ^30^ ^]^ on the use of phase‐changing materials,^[^ ^31^ ^]^ and on introducing a secondary heating loop to transfer heat for electricity generation or domestic hot water (DHW) demand.^[^ ^32^ ^]^

Hybrid photovoltaic‐thermal (PV‐T) concepts seek to exploit the synergistic nature of solar PV panels and ST collectors. Early examples were conceived aiming to cool PV modules and increase their electrical performance, however, the resulting thermal output raised an interest in further exploitation.^[^ ^32^ ^]^ Air has been used as a heat transfer fluid (or, coolant), but nowadays water‐based PV‐T collectors are more common, having gained popularity thanks to their improved performance.^[^ ^33^ ^]^ Most PV‐T collectors are typically aimed for use in domestic applications with a hot‐water delivery temperature up to 60–70 °C.^[^ ^34^ ^]^ The most recent research in this space focuses on PV‐T collector designs integrated with heat pipes, refrigerants, phase change materials (PCMs), as well as spectral splitting approaches, concentrating optics and nanoparticle doping, amongst other.^[^ ^35^ ^]^ Despite all of the above, hybrid PV‐T collector technology still requires significant roof space and, therefore, building integration of PV‐T systems is of particular interest. The prototype proposed in the present research adds value by exploring a semi‐transparent PV‐T window for building integration for the first time, which opens up the possibility of exploiting parts of the urban environment not previously available for simultaneous multi‐energy (electricity and hot water) generation. Transparency to the visible spectrum is a requirement for window integration, which for hybrid PV‐T collectors has not yet been explored.^[^ ^16^ ^]^

A review of window‐integrated solar energy technologies reveals room for improvement in the study of the performance of hybrid solutions for window integration in the scientific literature, with most existing studies mainly focusing either on solar electricity or heat generation,^[^ ^36^ ^]^ and less so on the cogeneration of both in buildings.^[^ ^37^ ^]^ A noteworthy study of a window‐integrated hybrid PV‐T system is that of Fieber,^[^ ^15^ ^]^ who considered a hybrid solar window composed of a series of blade‐shaped components (small PV‐T inserts and reflectors) forming a blinds‐like structure in which the reflectors concentrated sunlight onto the PV‐T collectors, consequently reducing the PV surface required for equal energy production compared to non‐concentrated PV collectors. The aperture of the blinds could be adjusted for room illumination control, while also inevitably affecting the electricity and heat provision. Technical challenges faced with this design include the diurnal variability of energy and the risk of concentrated radiation being reflected inside the building. Despite the efforts of this research group, window‐integrated PV‐T systems cannot be said to be a mature topic of research, and although some designs have been proposed in the form of schematics whose performance has been modelled,^[^ ^38^ ^]^ there is an even greater lack of supporting experimental data.

In this study, we designed, fabricated and tested a hybrid PV‐T window (PVTW), composed of a semi‐transparent PV module and a liquid‐based selectively‐absorptive ST absorber. An outdoor testing platform was established to measure simultaneously the thermal and electrical performance of the PVTW. The testing was conducted during typical summer days in London to assess the performance under different window inclination angles. Furthermore, the performance of the PVTW was compared with that of a standalone ST window (STW) to benchmark the performance of the hybrid design.

## 2 Experimental Section

### 2.1 Hybrid Photovoltaic‐Thermal Window Design

**Figure 1:**
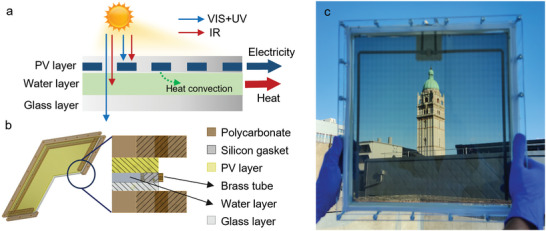
PVTW design: (a) conceptual design and working mechanism, (b) mechanical design, and (c) photograph of the PVTW at the South Kensington campus of Imperial College London.

The design and working principle of the PVTW is shown in **Figure** **1**a. Sunlight first strikes a semi‐transparent PV glass layer (supplied by Onyx Solar), which consists of an array of spaced a‐Si micro‐stripes. Only a part of the sunlight is absorbed by the PV layer for electricity generation, while the remaining portion is transmitted to the water layer below the PV layer. The water layer is nearly transparent to the visible (VIS) spectrum but can selectively absorb most of the infrared (IR) spectrum with wavelength longer than 1200 nm to generate thermal energy.^[^ ^39^ ^]^ The heat in the PV cells can be recovered by the flowing water via heat convection. The glass layer of the PVTW can effectively absorb part of the UV spectrum (less than 50% transmittance below 320 nm);^[^ ^40^ ^]^ in addition, the semi‐transparent PV layer only allows ≈1.5% UV transmission according to the manufacturer datasheet.^[^ ^41^ ^]^ Based on this working mechanism, the mechanical design illustrated in Figure 1b was conceived. Two polycarbonate frames were employed to clamp a standard low‐iron glass and the semi‐transparent PV layer together. A thick silicon gasket running along the perimeter between the PV layer and the bottom glass sheet was used to create a 4‐mm‐thick water layer. Two brass tubes puncturing the gaskets represent the inlet and outlet gates, which are placed in opposite corners. Finally, the photograph of the PVTW displaying Imperial College London's campus in the background is shown in Figure 1c. The PVTW looks semi‐transparent as a part of visible light can pass through the PV layer, the water layer and the bottom glass. The bold wires in the PVTW are the positive and negative wires for connecting the solar cells.

The geometric, electrical, and optical parameters of the PVTW are listed in **Table** **1**. The average transmittance of the semi‐transparent PV layer is provided by the manufacturer. The water mass flow rate in this study was carefully adjusted prior to, and then held constant during each run, to ensure the outlet temperature of the water reached ≈50 °C at midday, which is our target temperature for DHW applications. The bottom low‐iron glass was purchased from London Glass, with the same width and length as the PV module.

**Table 1:** Geometric, electrical and optical parameters of the PVTW.

Layers	Parameter/variable	Value
Semi‐transparent PV layer	Transmittance (*τ*_SPV_)	0.20 [‐]
	Reflectivity (*γ*_SPV_)	0.08 [‐]
	Width of effective area (*W*)	33 [cm]
	Length of effective area (*L*)	33 [cm]
Water layer	Transmittance (*τ*_w_)	0.90 [‐]
	Thickness/depth (*δ*_w_)	4 [mm]
	Inner diameter of the brass tube (*d*_i_)	3 [mm]
	Outer diameter of the brass tube (*d*_o_)	4 [mm]
Glass layer	Transmittance (*τ*_g_)	0.93 [‐]
	Thickness (*δ*_g_)	4 [mm]

The intrinsic multidisciplinary nature of the PVTW led to several material selection and engineering design challenges. In terms of the window design, selecting an appropriate semi‐transparent PV module to employ and ensuring efficient water sealing represented key issues. The design developed (as detailed above) not only allowed us to seal the PVTW successfully, but also to control accurately the depth of the water layer. To ensure semi‐transparency of the PV layer, we made use of an equally spaced number of a‐Si PV strips which allowed a portion of the incident light proportional to their covering factor to pass through.

**Figure 2:**
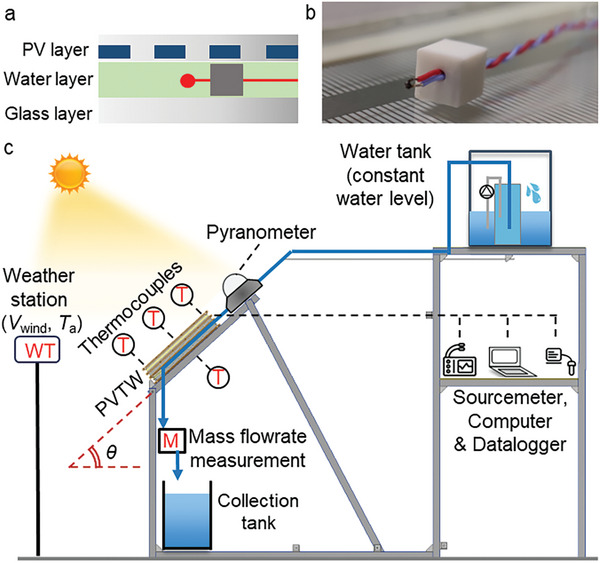
Measuring system used in the present work. (a) Conceptual solution used to suspend thermocouples in the bulk of the water flow, (b) photograph of the cubic rubber support used to hold the thermocouples, and (c) schematic of the measurement systems.

During testing, challenges were identified in controlling the flow rate and obtaining reliable temperature data for the water flow through the prototype. A procedure was established with a continuous flow rate measurement used to control the flow rate via a valve system, while rubber supports held the thermocouples suspended in the bulk of the water flow as shown in **Figure** **2**a,b.

Attention is also needed to ensure sufficient mixing within the water layer to avoid thermal stratification issues, which will exacerbate as the surface of the window increases. In future work, the stratification issue will be addressed by exploring more advanced water channel designs, such as using serpentine channels, which can improve flow distribution and reduce stratification.

### 2.2 Outdoor Testing System

The testing platform is shown in Figure 2c. The left portion of the structure supports the PVTW and the pyranometer (Kipp&Zone CMP 6) while offering the possibility of changing the inclination angle. The right portion of the system is composed of two shelves. The upper shelf is reserved to place a thermally insulated water tank as gravity is exploited for the water flow. The lower shelf is used to store the instrumentation, including a sourcemeter (Keithley 2460), a datalogger (PicoLog) and a computer. A weather station (Youshiko YC9800) is erected in the proximity of the PVTW to detect wind intensity and ambient temperature.

**Figure 3:**
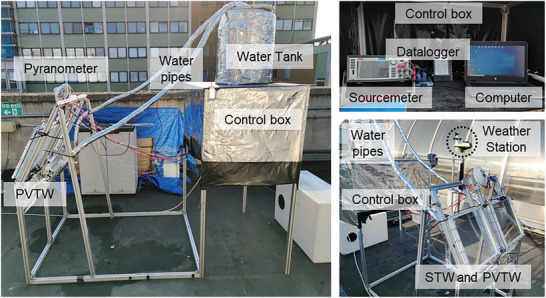
Photograph of the test facility at Imperial College London.

A valve is used to control the water mass flow rate, which is measured via a measuring cylinder and a high‐precision stopwatch; based on this measurement feedback, the inlet valve is appropriately (manually) adjusted to maintain a constant flow rate. A curve was generated to fit the measurement points mentioned above as a function of time which was used for our calculations. The water flow is driven by gravity, so keeping the water level in the feed tank constant is important for a stable mass flow. A secondary overflow chamber inside the feed tank is designed to provide a constant water level. The water only passes once through the PVTW. The water tank located at the end of the system is only used for collection purposes and it is not connected to the upper/supply tank. Once the lower tank becomes full, it is emptied manually. Three different inclination angles *θ*, as defined in Figure 2c, are investigated (*θ* = 30°, 60°, and 90°). Three thermocouples are distributed in the water layer, to measure the water temperature at the inlet, outlet and center of the PVTW. Supports are used to hold the thermocouples to measure water temperatures and avoid contact with the PV layer or the glass layer. An additional thermocouple is used to measure the ambient temperature. The sourcemeter is used to measure the current–voltage (*I*–*V*) curve of the PVTW to identify its maximum power point (MPP). This needs to constantly monitor the PVTW to ensure production of power at the MPP. Therefore, an in‐house algorithm (FP‐PV‐MPPT v1.0) was developed via the Test Script Builder (TSB) platform to instruct the sourcemeter at automatically tracking the MPP of the PVTW in transient outdoor environment. The FP‐PV‐MPPT v1.0 (suitable for Keithley sourcemeters) is available via the email address provided in the Acknowledgements for academic research purposes only. The test facility was located on the roof of the Roderic Hill Building in the South Kensington Campus of Imperial College London (51° 29′ 58.11″ N 0° 10' 41.7648″' W) and was oriented southwards. Testing was conducted between the 16th and the 23rd of July 2021. Photographs of the test facility in its testing environment are shown in **Figure** **3**.

In order to evaluate the performance of the PVTW, several parameters were calculated from the data. The PVTW electrical performance is characterized by the electrical efficiency:

(1) $$\eta_{\mathrm{el}}=\frac{P}{G\cdot A}\;$$

where *η*_el_ is the electrical efficiency, *P* the power produced by the PVTW (measured by the sourcemeter), *G* the solar irradiance (measured by the pyranometer), and *A* the PVTW surface area.

To further validate the test results, the PVTW electrical efficiency was also calculated separately by using information provided in the PV module manufacturer datasheet, and the relation:

(2) $$\eta_{\mathrm{el},\;m}=\left[1+\left(T_{\mathrm{PV}}-T_{\mathrm{ref}}\right)\cdot \beta \right]\;\cdot \eta_{0}$$

where *T*_PV_ is the average PV layer temperature, *η*_0_ is the electrical efficiency at *T*_ref_ = 25 °C, and *β* is the temperature coefficient of the PV cell (*η*_0_ = 3.4% and *β* = −0.19%/K according to the manufacturer datasheet).^[^ ^41^ ^]^ The PV layer temperature is a key parameter affecting electrical performance and from Equation (2) it is possible to estimate the PV efficiency over its entire operating temperature range (from −40 to 85 °C as specified in the datasheet).^[^ ^41^ ^]^ In case of warmer conditions, the operating temperature can be controlled so as not to surpass such threshold for DHW usage by increasing the flow rate through the water layer. This avoids operating the PV cell in conditions of very low electrical efficiency and increases lifetime (by reducing degradation and the risk of damage), while also increasing the thermal output of the system. The PV layer temperature *T*_PV_ was indirectly measured during the testing. Specifically, as the PV temperature distribution is not uniform, the PV layer temperature was inferred by averaging values from multiple thermocouples distributed over the PVTW.

The thermal efficiencies of the PVTW and STW were calculated by:

(3) $$\eta_{\mathrm{th}}=\frac{m\cdot c_{p}\cdot \left(T_{\mathrm{out}}-T_{\mathrm{in}}\right)}{G\cdot A}\;$$

where *ṁ* and *c*_p_ are the mass flow rate and specific heat capacity of the water flow. The inlet temperature *T*_in_ and outlet temperature *T*_out_ were measured via the thermocouples.

## 3 Results and Discussion

This section presents the thermal and electrical performance of the PVTW under real, outdoor environment testing conditions. For each case, we show the transient temperature distributions first, followed by transient distributions of electrical parameters (voltage, current, power). Electrical and thermal efficiencies are then calculated for different conditions. The mass flow rate of the water through the PVTW was set initially to 15 mL min^−1^, with deviations from this setting during testing accounted for by performing periodic flow rate measurements and using the actual flow rate value for our performance (e.g., *η*_th_) calculations.

### 3.1 Performance at 30° Inclination

The inclination angle of the PVTW was set to 30° to investigate the integration of this technology within skylights or glass roofs for housing or business purposes. The performance of the 30° inclined PVTW was tested on the 18th of July 2021.

**Figure 4:**
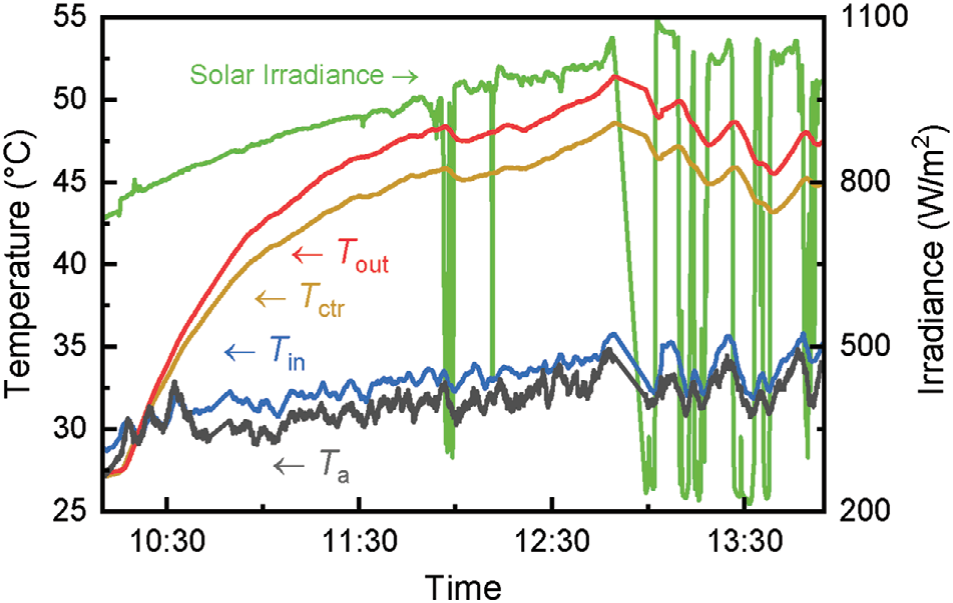
Temperature and solar irradiance (*θ* = 30°) profiles at the test location. Tests took place from ≈10:00 to 14:00 local time.

The inlet (*T*_in_), outlet (*T*_out_), and center (*T*_ctr_) temperature of the water channel from early morning until midday are presented in **Figure** **4**. The maximum ambient temperature (*T*_a_) detected reaches ≈34 °C, while the maximum solar irradiance reached ≈1100 W m^−2^ at midday. The solar irradiance kept increasing from ≈700 W m^−2^ at 10:00 and only occasionally fluctuated when clouds shaded the device. Despite the good thermal insulation of the water feed tank and the water feed pipe, ambient temperature still affected the inlet temperature due to the long residence time of water in the feed pipe. Both the center and outlet temperature showed a steep increase from 10:00 until reaching a more stable zone between 12:00–13:00. The relatively sudden changes in the outlet and middle temperatures are a result of solar irradiance variations affected by the sudden cloud shading. Despite the solar irradiance steeply decreasing from ≈950 to ≈300 W m^−2^ during 11:50–12:00, as shown in Figure 4, the relative change of the outlet temperature was small (a few °C) thanks to the PVTW's thermal inertia.

The outlet temperature is a key indicator of the quality of the PVTW's thermal output; on this typical summer day in London, and for the set water flow rate, the outlet temperature peaked at ≈50 °C, which is suitable for domestic hot water (DHW) use. Interestingly, the center temperature was close to the outlet temperature indicating that the PVTW was near stagnation conditions, and that ≈50 °C is close to the maximum temperature the PVTW can produce at the specific ambient conditions and operating parameters (e.g., water flow rate) used in the tests.

**Figure 5:**
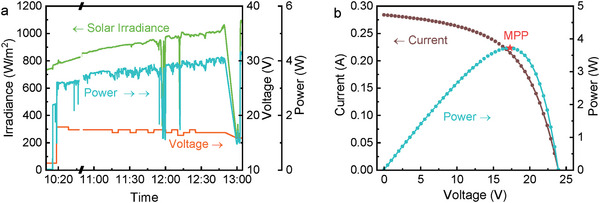
Electrical performance of the PVTW: (a) power and voltage profiles, and (b) *I–V* curve and MPP of the PVTW at 11:40 (*θ* = 30°).

The electrical performance indicators of the PVTW are presented in **Figure** **5**. Due to a sourcemeter malfunctioning, 12 min (10:38–10:50) of detections have been omitted from the graph to increase clarity and legitimacy; this does not affect the overall electrical performance evaluation. Power production fluctuated while increasing from 3 to 4 W throughout the morning. A higher solar irradiance results in higher power output. Cloud shadowing significantly affects electricity production as visible from the drops in correspondence of the solar irradiance variation ≈12:00. The sourcemeter quickly (7 s) sweeps the working voltage and generates an *I–V* curve to identify the MPP. The voltage across the PVTW is timely adjusted by the sourcemeter to ensure the PV cells are always working at the MPP condition. As an example, the *I–V* curve plotted at 11:40 is shown in Figure 5b. The voltage values in Figure 5a represent the optimal working voltages, as imposed by the sourcemeter, to achieve maximum power production by the PVTW in transient solar irradiance. It is interesting to note that the optimal voltage is almost constant (≈17 V) throughout the whole exposure period.

**Figure 6:**
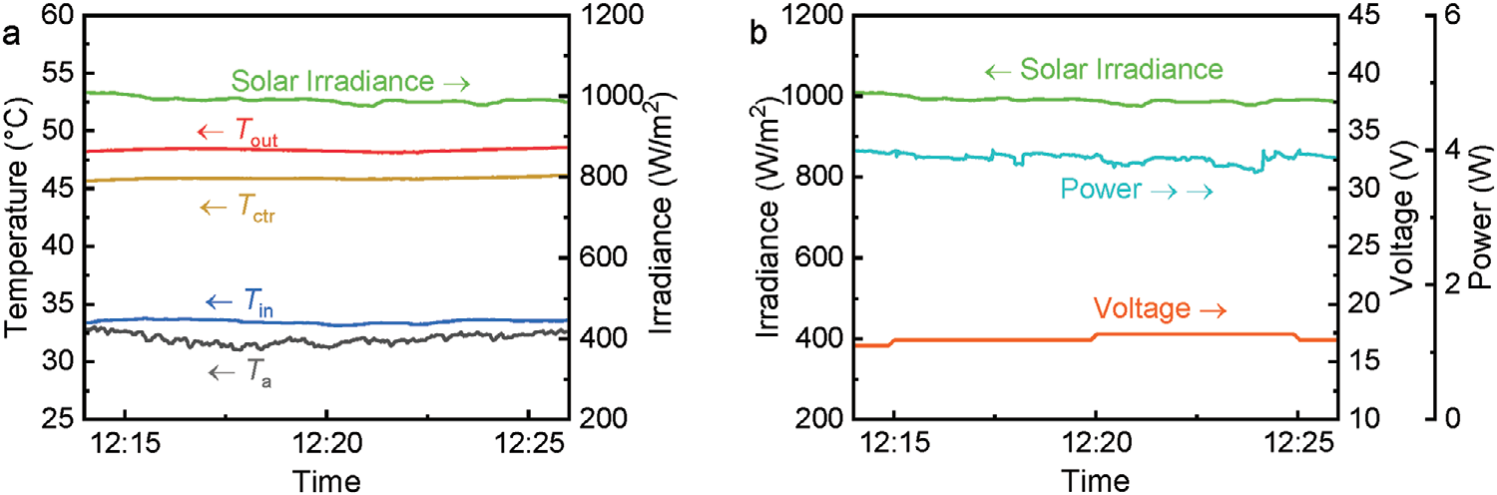
Thermal and electrical performance over a selected time period (12:15–12:25, *θ* = 30°): (a) temperature profiles, and (b) power and voltage profiles.

**Figure** **6** shows the thermal and electrical performance in a quasi‐steady‐state period (12:15–12:25) extracted from Figures 4 and 5. Throughout this study, quasi‐steady‐state conditions are determined in a set of statistically convergent data that vary by less than 10% of the variable's own value over 10 min. Maintaining an exact flow rate during the experiments was challenging, so to ensure greater accuracy in our performance calculations, the flow rate was periodically monitored throughout the testing period, and the real‐time measured values were used in calculating *η*_th_. Since the flow rate fluctuated slightly during operation, the average flow rate for the reference period (12:00 to 12:10) was 11.2 mL min^−1^, which was lower than the initial setting of 15 mL min^−1^. As shown in Figure 6a, water was heated up by ≈15 °C, with more than 80% of this heating occurring in the inlet section (i.e., from the inlet to the center part of PVTW), indicating that the PVTW had reached a stagnation heating situation: further decreasing the mass flow rate would have had a slight impact on increasing the outlet temperature.

**Figure 7:**
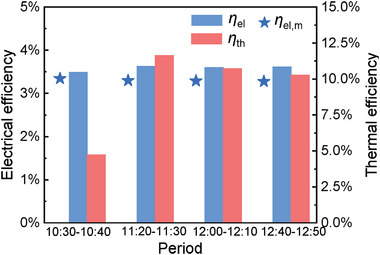
Thermal and electrical efficiencies of the PVTW at different times (*θ* = 30°). The measured electrical efficiency is validated against manufacturer data.

The efficiencies of the PVTW (*θ* = 30°) are reported in **Figure** **7**. This includes the measured electrical and thermal efficiencies (*η*_el_ and *η*_th_) as well as the expected electrical efficiency (*η*_th,m_) according to the manufacturer in the same PV operation temperature. The presented efficiency results are averaged over 10‐min periods starting from 4 different times (10:30, 11:20, 12:00, 12:40) of the day to better depict the performance from start‐up to quasi‐steady‐state.

The electrical efficiency of the PVTW barely changed in all the analyzed periods and kept stable at 3.6%. However, thermal efficiency changed significantly from the morning to the midday, being lower at 10:30 (4.7%) to then stabilize from 11:20 onwards (over 10%). Upon irradiation, electricity is produced instantaneously; the thermal response, on the other hand, has a significant delay time caused by the high heat capacity of the water channel. Therefore, the electrical efficiency keeps stable while the thermal efficiency takes longer to reach stability. The detected electrical efficiency agrees well with the manufacturer's prediction, which strengthens the validity of our measuring system. Undoubtedly, the heating up of the PVTW from morning to midday negatively affected the electrical efficiency; however, the minor PV temperature build‐up from 10:30 to 12:00 (<10 °C) together with the reduced impact of temperature on this specific PV cell (*β* = −0.19%/K) allowed for the increase in solar irradiance to balance the expected electrical efficiency drop. The overall efficiency (electrical + thermal) of the PVTW reached 14.3% at midday, which is ≈4 times the standalone PV efficiency (3.6%).

### 3.2 Performance at 90° Inclination

To assess the feasibility for vertical window integration of the PVTW, the inclination angle was increased to 90°. The performance of the vertical PVTW was tested on the 21st of July 2021.

**Figure 8:**
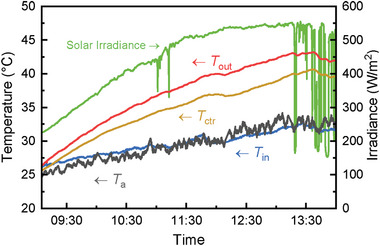
Temperature profiles and solar irradiance (*θ* = 90°).

The temperature detection results, summarized in **Figure** **8**, exhibit a significant resemblance with the ones from the 30° test, sharing an initial growth until peaking at midday and starting to decline. The solar irradiance trend is consistent with the data previously presented in Figure 4; however, a crucial difference lies in the magnitude of the detections. Peaking only just above 500 W m^−2^, the 90° inclination results in a 45% reduction compared to the 30° one. The reduced solar irradiation is one of the key challenges to tackle while designing a vertical solar harvester. As a result of the reduced irradiance, the maximum outlet water temperature of the 90° inclination PVTW was only ≈42 °C, which is ≈7 °C lower than that of the 30° inclination PVTW.

**Figure 9:**
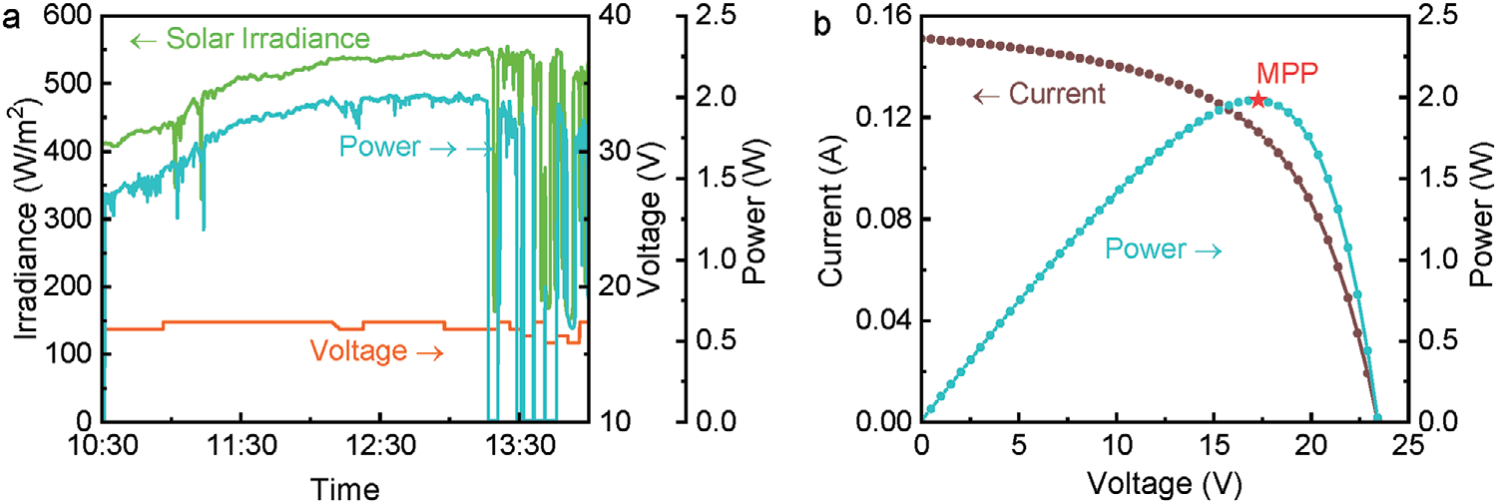
Electrical performance of the PVTW (*θ* = 90°): (a) power and voltage profiles, and (b) *I–V* curve and MPP of the PVTW at 12:30.

The electrical performance indicators of the PVTW are presented in **Figure** **9**. Differently from the thermal behavior, the electrical performance of the device was significantly influenced by the drop in irradiance, as Figure 9 reveals. Power levels show the same degree of stability of the inclined roof application (*θ* = 30°), but with peak production at ≈2 W; the same 45% drop endured by solar irradiance is therefore visible. Voltage levels, on the other hand, are barely distinguishable from the 30° inclination case because of the same *I–V* curve shape, only being shifted to lower current values. This proves the consistency of the ambient conditions upon different days and inclinations. The electrical performance is instantaneously affected by solar irradiance. On the other hand, the thermal output has a longer response due to the components’ heat capacities. The 90° inclination PVTW had a more durable and less steep increase in temperatures compared to the 30° inclination, due to the lower solar irradiance.

**Figure 10:**
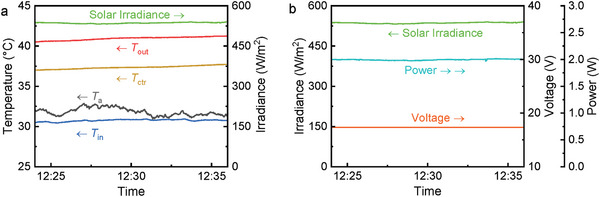
Thermal and electrical performance in a selected period (12:25–12:35, *θ* = 90°): (a) temperature profiles, and (b) power and voltage profiles.

As per previously, a close‐up analysis of a subsection of steady‐state data is presented in **Figure** **10**. A 10‐min window (12:25–12:35) of steady‐state conditions was identified with only slight fluctuation over the ambient temperature. The inlet‐outlet temperature difference is ≈10 °C. Also in this case, the steady‐state conditions coincide with peak power production from the device.

**Figure 11:**
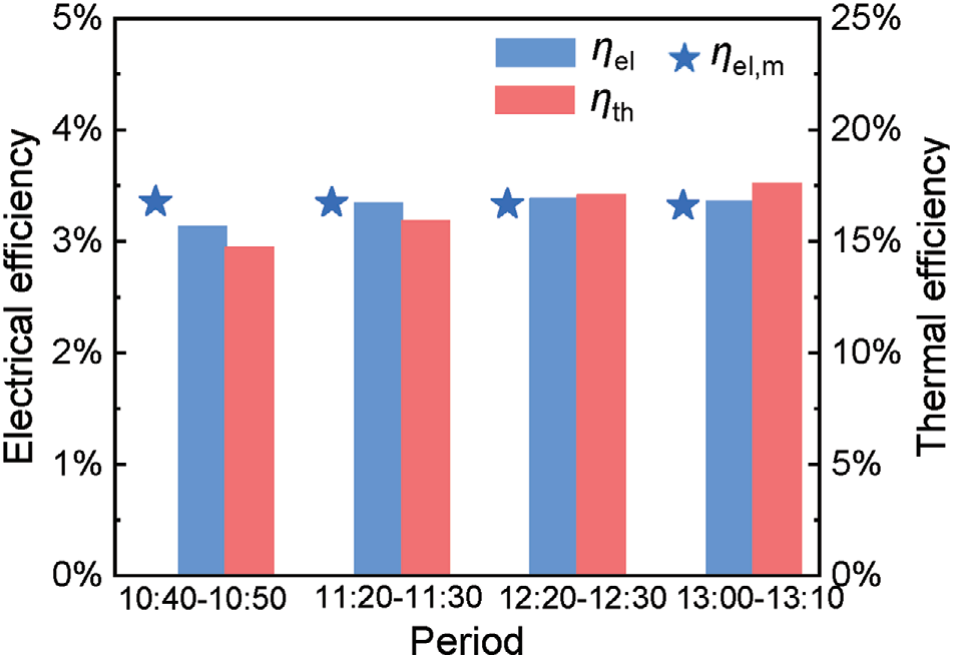
Thermal and electrical efficiencies of the PVTW at different times (*θ* = 90°). The measured electrical efficiency is validated against manufacturer data.

To assess the viability of the PVTW for vertical applications it is crucial to evaluate the effect of the reduced irradiance on te efficiency levels. The highlighted drops in the device's electrical and thermal output do not imply a reduction in its efficiency as the energy entering the system diminished as well. **Figure** **11** collects the efficiency results averaged over 10‐min periods starting from 4 different times (10:40, 11:20, 12:20, 13:00). The electrical efficiency of the PVTW is slightly reduced, reaching a value of 3.3%; the data's legitimacy is once again strengthened by the consistency of the electrical efficiency detections with the manufacturer specifications. Thermal efficiency shows a similar growth trend, increasing from the morning until peaking around midday just below 17.6%. When comparing the values with the 30° inclination ones, only an 0.3% absolute reduction in electrical efficiency occurs. Thermal efficiency on the other hand was drastically improved when *θ* = 90° with values soaring from 10.7% to over 17.6%. Overall efficiency of the system had therefore improved from 14.3% to 20.9%, due to the decrease in irradiance, and therefore in energy, entering the PVTW system.

### 3.3 Impact of Inclination Angle on Performance

**Figure 12:**
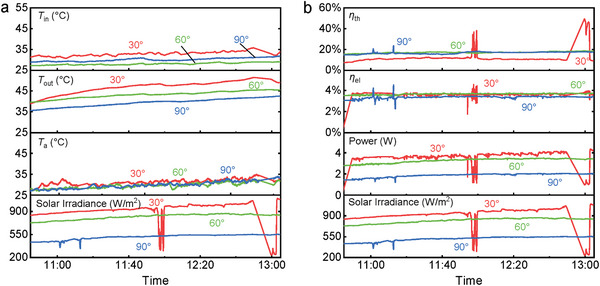
Impact of inclination angle on: (a) thermal, and (b) electrical performance.

To further highlight the impact of the inclination angle *θ* on the performance, the results of 30°, 60°, and 90° inclinations are compared in **Figure** **12**. Measurements were conducted on different days of the same week where ambient conditions were consistent as the detection shows.

The impact on solar irradiance was significant with its value being almost halved in 90° inclination compared to 30° one. Solar irradiance shows a non‐linear variability with the inclination angle *θ*. Power production fully aligns with the solar irradiance trend as its value is almost halved moving from 30° to 90° inclination. The intermediate angle (60°) power production performance is more similar to the 30° one, again due to the non‐linear dependence between the two. Despite this drop in power, electrical efficiency is almost unchanged as the irradiance reduction balances out for the inferior power production. Focusing on the temperature results, an outlet temperature drop of 7–8 °C can be found between 30° and 90°. However, a difference of 2–3 °C already exists between the inlet temperatures of these inclinations decreasing the effective difference to only ≈5 °C. The 30° inclination PVTW has the highest operating temperature, and thus has the lowest thermal efficiency due to the highest heat loss. As per before, the irradiance impact on the electric behavior is more significant than the thermal one due to the significantly different response times of the two.

**Figure 13:**
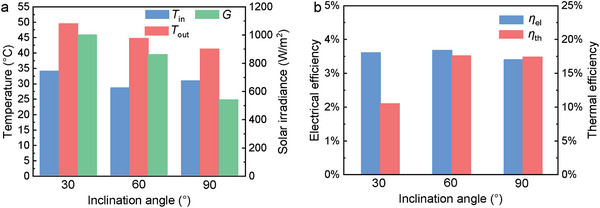
PVTW performance comparisons for different inclination angles at 12:30–12:40: (a) inlet/outlet temperatures and solar irradiances, and (b) electrical and thermal efficiencies.

A summary of key performance indicators achieved by the PVTW for different inclination angles between 12:30 and 12:40 is shown in **Figure** **13**. During this period, the PVTW reached quasi‐steady‐state working conditions for all inclination angles. As illustrated in Figure 13a, the solar irradiance received on the PVTW decreases at higher inclination angles, resulting in a corresponding decrease in outlet temperatures. While the electrical efficiencies of the PVTW at different inclination angles are similar due to the comparable reduction in irradiation and power production, the panel with an inclination angle of 30° exhibits the lowest thermal efficiency. This occurs because the PVTW with a 30° inclination reaches a stagnation temperature condition, as evidenced by Figure 4, which shows the outlet temperature closely matching the center position temperature, leading to significant heat loss. In future research, the water mass flow rate for the PVTW with a 30° inclination should be increased to enhance thermal performance.

### 3.4 Comparison of Performance to a Solar Thermal Window

**Figure 14:**
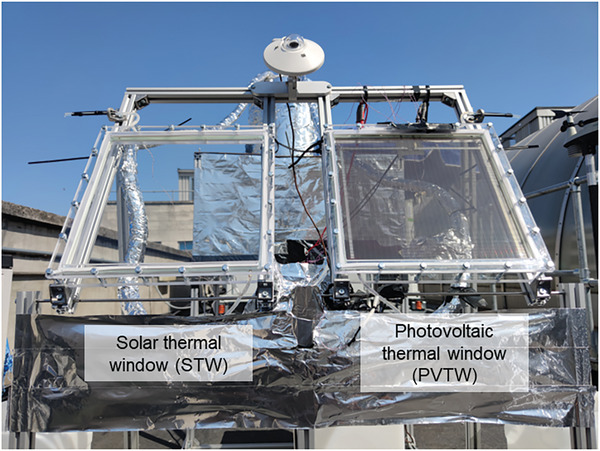
Photograph of the photovoltaic thermal window (PVTW) on the right and the solar thermal window (STW) on the left.

In an attempt to compare and quantify any potential advantages of hybridization relative to a dedicated solar thermal window (STW), i.e., without the integration of a PV module, a STW was fabricated and tested. This device is identical to the PVTW in every design aspect except for substituting the PV layer with a glass layer; therefore, it is composed of two glass layers with an average transmittance of ≈0.93, and a water layer with an average transmittance of ≈0.90 over the 280–1350 nm spectral region^[^ ^42^ ^]^ (see Table 1). This transforms the device into a standalone STW harvester with an overall transmittance of 0.78 (two glass layers, and one water layer), which can be seen placed next to a PVTW with an overall transmittance of 0.17 (one glass layer, one water layer, and a PV layer with a lower transmittance of 0.20) in **Figure** **14**.

**Figure 15:**
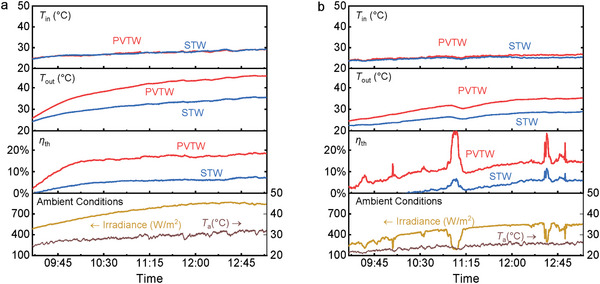
PVTW and STW performance comparisons in tests conducted on consecutive days at different inclination angles: (a) 60°, and (b) 90°.

The two devices were tested simultaneously with equal conditions for two consecutive days; **Figure** **15** summarizes the results obtained with a 60° and 90° inclination angle (*θ*). Simultaneous testing ensures the devices to be exposed to identical ambient conditions. Additionally, as inlet temperatures coincide fully, it is possible to state that any different result between the PVTW and the STW is solely due to the hybrid working conditions over the standalone ones. For the 60° inclination, a huge discrepancy in outlet temperature occurred with the PVTW peaking at over 44 °C while the STW barely reaching 34 °C. In both cases, the device is likely to be used in addition to a traditional heating system to lighten its load and primary energy consumption and to ensure that the demand can be met despite the intermittent and variable nature of solar energy. Nonetheless, this 10 °C variation highly influences the dispatchability of the thermal output for DHW use, allowing a significant reduction in the boiler load or even for the direct use of water. Focusing on data extracted from 10:00 onwards, when both devices had already experienced most of their transient conditions, the thermal efficiency data clearly show the advantages of the hybrid PVTW (16.7% between 10:00 and 13:10) over the STW alone (5.8% between 11:00 and 12:00). Of note is that the PVTW also has an electrical efficiency of ≈3.0–3.6%, which is not shown in Figure 15.

Apart from a reduction in the variables magnitude consequent to the irradiance drop, similar behavior was encountered when investigating the devices performance upon vertical inclination (90°). Inlet temperature was almost identical for the two devices and outlet temperature resulted higher for the hybrid PVTW. The difference in outlet temperature is reduced when *θ* = 90°, with ≈35 °C for the PVTW and ≈28 °C for the STW. However, this still shows a significant improvement for the hybrid PVTW. The thermal efficiency variation between the two devices is identical to the 60° inclination, but the curves are shifted to lower values for the reduced temperature and irradiance. In the early morning, the efficiency of the STW was even negative. This because when irradiation is low, the heat loss of the STW is even higher than the absorbed solar energy. Focusing on efficiency values from 10:00 onwards as per before, the PVTW showed a 13.3% thermal efficiency whilst the STW 3.4%.

**Figure 16:**
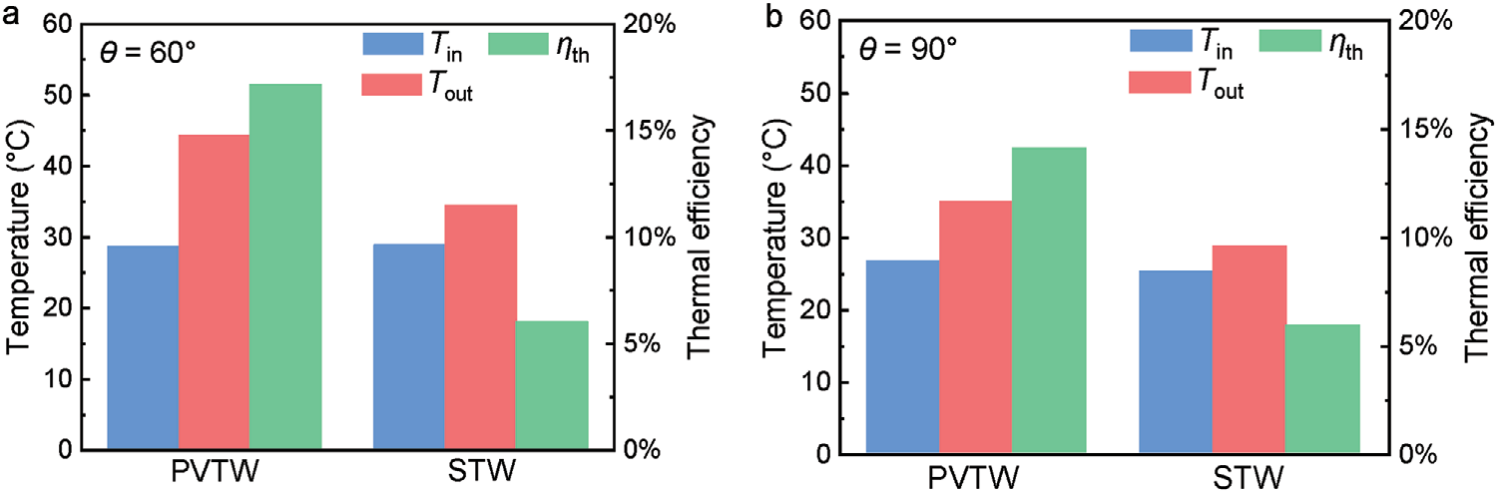
PVTW and STW thermal performance comparisons at 12:20–12:30: (a) inlet/outlet temperatures and thermal efficiencies when the inclination angle is 60°, and (b) inlet/outlet temperatures and thermal efficiencies when the inclination angle is 90°.

A comparison between the PVTW and STW key performance indicators at various inclination angles between 12:20 and 12:30 is shown in **Figure** **16**. During this period, both the PVTW and STW achieved quasi‐steady‐state working conditions with similar water inlet temperatures. At an inclination angle of 60°, the PVTW exhibited a temperature difference of 15.7 °C between its inlet and outlet, which is significantly higher than the 5.5 °C attained in the STW. The thermal efficiencies of the PVTW and STW at this angle were 17.1% and 6.0% respectively, as shown in Figure 16a. When the inclination angle was increased to 90°, the temperature difference between the outlet and inlet in the PVTW was 8.3 °C, again surpassing the 3.4 °C difference in the STW. The corresponding thermal efficiencies were 14.1% for the PVTW and 5.9% for the STW, as shown in Figure 16b. These results indicate that, beyond providing an electrical output, the thermal efficiency of the PVTW consistently exceeded that of the STW.

The results discussed above underline the synergistic nature of the PVTW design, which exploits the otherwise unutilized heat in the PV layer and in doing so outperforms the non‐hybrid STW both in terms of electrical and thermal production. Specifically, the comparison with the STW in Figure 16 shows that the thermal performance of the PVTW benefits from making use of the unutilized heat in the PV layer, both resulting from greater direct solar absorption by the PV layer and by recovering the thermal energy generated within the PV material by the electricity generation process. As a matter of fact, the experimental results highlighted that solar absorption by the water layer in the STW produces a poor thermal output in testing conditions, such that even the thermal output of the PVTW is considerably better than a dedicated STW.

A case could be made that the selection of a different fluid (or doping) or a different glass layer could lead to better performance. However, this is outside the scope of this work, which presents the comparison with a STW solely to show that the thermal performance of the PVTW benefits greatly by recovering and making use of the PV layer heat.

Based on our experimental results, for a PVTW with an area of 1 m^2^ at a 30° inclination and a well‐insulated water storage tank with a volume of 1 L, we would need 10.6 min at midday to fill the tank with 50 °C hot water (based on a flow rate of 0.17 mL s^−1^, as measured between 12:40 and 13:00 when the PVTW was exposed at 30° inclination and reached 50 °C). Additionally, it is noteworthy that during operation, the PVTW would also generate additional electricity with an average efficiency of 3.5%, whereas the STW solely produces heat.

To understand the potential impact of the PVTW in meeting a building's thermal energy needs, we can estimate the PVTW surface required to meet the hot‐water demand of a typical three‐bedroom terraced house in London, UK, occupied by 2 adults and 2 children. Between 11:30 and 13:30, which overlaps with the duration of the present tests, the instantaneous (average) hot‐water consumption of the above‐mentioned reference household drops from 6.4 to 4.8 L h^−1^ at 50 °C.^[^ ^13^ ^]^ Using an energy balance while assuming conditions similar to those experienced in the tests in Figure 4 and based on the PVTW thermal performance summary in Figure 7, an overall PVTW surface of no more than ≈1.2 m^2^ at a 30° inclination would be needed to meet this demand instantaneously, with a controlled, time‐variable (e.g., 0.17 mL s^−1^ from 12:40 to 13:00) flow rate needed to ensure the hot water delivered by the PVTW reaches the desired temperature over this time period. Furthermore, the mean daily household hot‐water consumption in England is 122 L day^−1^.^[^ ^13^ ^]^ Assuming a wider system with a hot‐water storage tank, and again based on the conditions and performance in Figures 4 and 7, we estimate an overall PVTW surface of ≈2.8 m^2^ at the same inclination would be needed to meet this entire daily demand without the need for a backup boiler.

In a real application, a loss in dispatchability will result due to solar intermittency and variability, so it is important to emphasize that this technology would play a role by reducing the load of traditional water heating systems that would act as backup rather than entirely substituting them. The estimates provided here suggest that a relatively limited area of PVTWs would significantly reduce the load of a gas‐fired backup boiler in the considered case study.

## 4 Conclusion

This study marks an important step forward in renewable energy integration within urban architectures, as demonstrated by the successful development and testing of a building‐integrated hybrid photovoltaic‐thermal window (PVTW). Our experimental results in London (maximum ambient temperature ≈34 °C, solar irradiance ≈1100 W m^−2^ at midday) indicate that this hybrid solar technology can effectively generate both electricity and heat, a dual benefit that is particularly valuable in urban areas where space is at a premium.

The PVTW combines a semi‐transparent photovoltaic layer with a selectively‐absorptive liquid‐based thermal absorber, achieving efficiencies of 3.6% in electricity and 10.7% in heat generation at a 30° inclination. The ability to produce ≈50 °C hot water makes it appropriate for domestic applications, while its electricity generation supports the building's energy needs. Adjusting the PVTW's inclination angle from 30° to 90° demonstrates the importance of orientation in system performance, with changes in output temperature and thermal efficiency observed. Compared to a standalone solar‐thermal window (STW), the PVTW not only provides higher temperature hot water but also shows a 10% absolute increase in thermal efficiency, along with electricity generation.

Future research should focus on evaluating the PVTW's performance across a broader range of weather conditions, particularly by analyzing the impact of key parameters such as irradiance and ambient temperature. Such investigations would help verify (or not) our hypothesis that the system's behavior in colder, cloudier and lower irradiance environments may mirror the patterns observed in our current examples after 13:00, when intermittent cloud cover led to fluctuations in the electrical output. Under these conditions, the output would be similarly variable to the behavior observed between 9:00 and 10:00 in our experiments.

Crucially, the aim of this technology is not to replace traditional opaque PV panels and ST collectors but to complement them. By transforming an additional portion of the building envelope into a multipurpose energy producer, the PVTW represents a strategic addition to sustainable urban design. It is important to emphasize the distinct target and application of the PVTW system compared to traditional solar systems. Conventional, high‐efficiency PV and ST systems are typically designed for installation on roofs or (less often) building façades or other walls, a resource that is often limited in densely populated urban areas. PVTW systems, on the other hand, are designed for building integration as window replacements, which provides additional energy‐generating surfaces without competing for roof space. Therefore, the primary aim of a PVTW system is not solely to maximize energy efficiency but to also meet the daylighting needs in buildings, while simultaneously generating electricity and heat, but also reducing space heating, and the associated energy consumption needed for cooling.

Our research paves the way for optimizing the structural design of window‐integrated distributed energy solutions, particularly in anticipation of expected short‐term advancements in selectively transparent PV materials. The installation of PVTWs comes with additional costs which should be considered relative to the economic and environmental value of the thermal and electrical outputs of the device. Window frames would require upgrade to accommodate the increased weight, and additional piping to/from the existing hot water supply systems would be needed together with supplemental pumping to circulate through the device.

The specific weight of the PV layer is 16.7 kg m^−2^ as specified by the manufacturer and the specific weight of 4‐mm‐thick low‐iron glass is 10 kg m^−2^. With the addition of 4 kg m^−2^ from the 4‐mm‐thick water layer, we can calculate the weight of the PVTW to be ≈30 kg m^−2^ without considering the frame, which is assumed to be unchanged. Compared to standard thin (4/20/4 mm) double‐glazed windows, which weigh 20 kg m^−2^, a ≈50% increase in weight is expected, however, compared to standard medium‐thickness (6/16/6 mm) double‐glazed windows, which weigh 30 kg m^−2^, we do not expect a notable increase in weight. Any increase will have to be accounted for when designing an appropriate frame to sustain the increased weight, while also granting access for water circulation loops and for connection cables.

For a fully integrated system in a real‐world building environment, this design would connect to the existing hot water piping system, utilizing pressurized water already present in the system. Therefore, the pressure required to operate the device would be negligible compared to the pressure levels typically found in the water mains. It is realistic to assume that with appropriate design, the device can operate effectively with gravity, as demonstrated in our test environment. Additionally, the system could operate thermosiphonically, a principle commonly employed in traditional solar water heating systems, which allows fluid circulation based on temperature‐induced density differences, further reducing the need for active pumping. Our hope is that this research will motivate further investigations on solutions that can increase the performance and reduce the relative cost of the PVTW to the point that an economic benefit is realized.

The proposed PVTW design can be referred to as thermally‐coupled, meaning that the water and PV layers operate at similar temperatures. This solution, despite being less complex, requires a trade‐off between increasing the PV electrical output, which increases at a lower temperature, and the quality of the thermal output, which improves at higher temperatures. A more advanced design utilizing spectral‐splitting methodology can be considered in future work.

Current research efforts by numerous companies are geared toward achieving spectrally selective transparency, opening up new possibilities for window integration. Variables such as water‐layer thickness (depth), inlet‐outlet structure, and flow rate of PVTW can be optimized for enhanced performance.^[^ ^31^ ^]^ The data from our experimental campaign is invaluable for validating simulation models and providing optimization strategies. Furthermore, such an investigation enables the exploration of PVTW designs, preparing us for the future exploitation of transparent PV materials in hybrid solar energy harvesting. As these technologies mature, our findings will be instrumental in developing the necessary support equipment and systems, ensuring that urban buildings can effectively contribute to sustainable energy solutions.

In conclusion, the PVTW demonstrates a promising integration of solar energy harvesting into urban environments. As cities increasingly adopt glass‐based architectures, the potential for these buildings to contribute to energy generation becomes more significant. Our study underscores the transformative potential of PVTW in the sustainable energy landscape.

## Conflict of Interest

The authors declare no conflict of interest.

## Data Availability

The data that support the findings of this study are available from the corresponding author upon reasonable request.
